# A randomised, phase II study of nintedanib or sunitinib in previously untreated patients with advanced renal cell cancer: 3-year results

**DOI:** 10.1038/bjc.2015.313

**Published:** 2015-10-08

**Authors:** T Eisen, A-B Loembé, Y Shparyk, N MacLeod, R J Jones, M Mazurkiewicz, G Temple, H Dressler, I Bondarenko

**Affiliations:** 1 Department of Oncology, Cambridge University Health Partners, Addenbrooke's Hospital, Cambridge, UK; 2 Medical Department, Boehringer Ingelheim B.V., Alkmaar, The Netherlands; 3 Department of Chemotherapy, Lviv State Oncology Regional Treatment and Diagnostic Centre, Lviv, Ukraine; 4 Cancer Research UK Clinical Research Unit, Beatson West of Scotland Cancer Centre, Glasgow, UK; 5 Centrum Onkologii Ziemi Lubelskiej, Lublin, Poland; 6 Medical Department, Boehringer Ingelheim Ltd., Bracknell, UK; 7 Global Pharmacovigilance, Boehringer Ingelheim Pharma GmbH, Ingelheim, Germany; 8 Oncology Department, Dnipropetrovsk State Medical Academy, Clinical Hospital #4, Dnipropetrovsk, Ukraine

**Keywords:** Angiogenesis inhibitors, carcinoma, kidney, nintedanib, receptors, fibroblast growth factors, sunitinib

## Abstract

**Background:**

This exploratory study evaluated the safety/efficacy of nintedanib or sunitinib as first-line therapy in patients with advanced renal cell carcinoma (RCC).

**Methods:**

Ninety-six patients were randomised (2:1) to either nintedanib (200 mg twice daily) or sunitinib (50 mg kg^−1^ once daily (4 weeks on treatment; 2 weeks off)). Primary endpoint was progression-free survival (PFS) at 9 months. *P*-values reported are descriptive only; the study was not powered for such comparisons.

**Results:**

Progression-free survival at 9 months was comparable between nintedanib and sunitinib (43.1% *vs* 45.2%, respectively; *P*=0.85). Median PFS was 8.4 months in each group (hazard ratio (HR), 1.12; 95% confidence interval (CI): 0.70–1.80; *P*=0.64). Median overall survival was 20.4 and 21.2 months for nintedanib and sunitinib, respectively (HR, 0.92; 95% CI: 0.54–1.56; *P*=0.76). Overall incidence of any grade adverse events (AEs) was comparable (90.6% *vs* 93.8%); AEs grade ⩾3 were lower with nintedanib than sunitinib (48.4% *vs* 59.4%). Nintedanib was associated with lower incidences of some AEs typical of antiangiogenic tyrosine kinase inhibitors (TKIs): hypertension, hypothyroidism, hand–foot syndrome, cardiac disorders and haematological abnormalities.

**Conclusions:**

In patients with advanced RCC, nintedanib has promising efficacy and similar tolerability to sunitinib, and a manageable safety profile with fewer TKI-associated AEs.

Over the past decade, antiangiogenic agents have become established as effective treatment options for patients with advanced renal cell carcinoma (RCC) (Conti *et al*, 2013). The currently approved tyrosine kinase inhibitors (TKIs), including sunitinib, sorafenib, pazopanib and axitinib, block tumour angiogenesis through inhibition of proangiogenic signalling receptors, such as vascular endothelial growth factor receptors (VEGFRs) and platelet-derived growth factor receptor-*α* (PDGFR-*α*; Bukowski, 2012; Conti *et al*, 2013). Sunitinib is one of the current standard-of-care options for treatment-naive patients with metastatic RCC, targeting VEGFR-1-3 and PDGFR-*α*/*β* (Pfizer Ltd, 2014).

Despite the efficacy of TKIs in patients with advanced RCC, disease progression occurs within a median of 9–12 months after treatment initiation (Hutson *et al*, 2008; Motzer *et al*, 2009; Sternberg *et al*, 2010). Prolonged treatment with some TKIs has also been associated with significant adverse events (AEs), including QT prolongation, cardiac toxicity and hypertension (Di Lorenzo *et al*, 2011; Ravaud, 2011; Ghatalia *et al*, 2015). In the pivotal Phase III study of sunitinib in RCC, 19% of patients discontinued sunitinib due to AEs (Motzer *et al*, 2009). Evidence demonstrates that sunitinib dose reductions or discontinuations are correlated with shorter survival for patients with advanced RCC (Porta *et al*, 2014). New therapies that provide a survival benefit with a manageable safety profile are needed.

Nintedanib is an orally available triple angiokinase inhibitor that specifically inhibits VEGFR-1-3, PDGFR-*α*/*β* and fibroblast growth factor receptor (FGFR)-1-3, as well as FLT-3, RET and members of the Src family (Hilberg *et al*, 2008; Reck *et al*, 2014). Unlike other currently approved antiangiogenic therapies that inhibit signalling through VEGFR and/or PDGFR, nintedanib simultaneously inhibits three angiogenic signalling pathways. A growing body of evidence suggests an important role for FGFR signalling in acquired resistance to VEGFR inhibition (Casanovas *et al*, 2005). Furthermore, the role of both FGF and PDGF signalling in the development of anti-VEGF resistance is supported by the clinical observation that FGF and PDGF plasma levels increase prior to disease progression in patients receiving anti-VEGF therapy (Kopetz *et al*, 2010).

Nintedanib (200 mg twice daily (bid)) in combination with chemotherapy has demonstrated significant efficacy in two Phase III studies of advanced non-small cell lung cancer (NSCLC; Hanna *et al*, 2013; Reck *et al*, 2014) and a Phase III study of advanced ovarian cancer (Du Bois *et al*, 2013). Recent Phase II studies comparing nintedanib and sorafenib as first-line monotherapy in patients with hepatocellular carcinoma (HCC) showed similar efficacy between the treatments in both Asian and European patient populations (Cheng *et al*, 2015; Palmer *et al*, 2015) with a more favourable safety profile reported for nintedanib in Asian patients (Cheng *et al*, 2015). Nintedanib has also shown significant efficacy in two Phase III trials of patients with idiopathic pulmonary fibrosis (IPF), a progressive and severely debilitating lung disease (Richeldi *et al*, 2014). Nintedanib is currently approved in the European Union (EU) in combination with docetaxel for locally advanced, metastatic or locally recurrent NSCLC of adenocarcinoma histology after failure of first-line chemotherapy (Boehringer Ingelheim, 2014b). Nintedanib is also approved in the EU and the US as monotherapy for patients with IPF (Boehringer Ingelheim, 2014a).

Clinical experience with nintedanib monotherapy in RCC has been reported from a Phase I study of patients with various solid tumour types, including metastatic RCC (Mross *et al*, 2010). Of the 10 patients with advanced RCC in that study, one patient had a partial response to treatment and one patient with lung metastases had a complete response (Mross *et al*, 2010). Two patients with advanced RCC received nintedanib treatment for >1 year, and seven were treated for at least 5 months. Nintedanib demonstrated a generally manageable safety profile, with the most frequently observed AEs being gastrointestinal effects (i.e., nausea, vomiting and diarrhoea; Mross *et al*, 2010).

Following the promising Phase I data, the current Phase II study (ClinicalTrials.gov, NCT01024920; Study 1199.26) evaluated the safety and efficacy of nintedanib as first-line therapy for previously untreated patients with advanced RCC. The study design included co-primary safety and efficacy objectives. The co-primary safety data for the nintedanib group have been reported previously (Eisen *et al*, 2013) and here we report on the co-primary efficacy and additional efficacy and safety endpoints.

## Patients and methods

### Patients

This study was conducted at 13 centres in five countries (Hungary, Poland, Romania, UK and Ukraine). Adults (age ⩾18 years) with a histologically confirmed diagnosis of unresectable or metastatic RCC with clear cell component, and measurable disease according to Response Evaluation Criteria in Solid Tumors (RECIST) version 1.1 were eligible for inclusion (Supplementary Table S1; Eisenhauer *et al*, 2009; Eisen *et al*, 2013).

All patients provided written informed consent. The clinical trial protocol was approved by the local and national independent ethics committees for each trial centre. The study was conducted in accordance with the International Conference on Harmonisation of Technical Requirements for Registration of Pharmaceuticals for Human Use (ICH) Harmonized Tripartite Guideline on Good Clinical Research Practice (1997) and with the principles of the Declaration of Helsinki.

### Study design and treatments

This randomised, multicentre, open-label, parallel-group Phase II study evaluated the safety and efficacy of nintedanib. The primary objective was to investigate the cardiac safety of nintedanib in terms of its effect on patients' QT interval, as reported previously (Eisen *et al*, 2013). The co-primary objective was to compare the efficacy and safety of nintedanib *vs* sunitinib ahead of potential Phase III clinical evaluation. However, this comparison was exploratory, and the study was not powered to allow a formal statistical comparison between the treatment arms.

Given the exploratory nature of the treatment comparison, patients were randomised in a 2 : 1 ratio to receive either oral nintedanib (200 mg bid) continuously in 4-week cycles or oral sunitinib (50 mg kg^−1^) once daily in 6-week cycles (4 weeks of sunitinib followed by 2 weeks without treatment). Randomisation was performed using a telephone Interactive Voice/Web-based Randomization System (IVRS/IWRS). Randomisation was stratified according to each patient's Memorial Sloan-Kettering Cancer Center (MSKCC) risk score (favourable/intermediate *vs* poor) (Motzer *et al*, 1999) and prior nephrectomy for RCC (yes *vs* no). Patients were treated until disease progression (according to RECIST version 1.1), death, unacceptable AEs, or withdrawal of consent for any other reasons. Two dose-reduction levels were available for patients experiencing drug-related AEs: 150 and 100 mg bid for nintedanib and 37.5 and 25 mg/kg once daily for sunitinib. Dose reductions in the nintedanib group were indicated for patients with diarrhoea grade 2 for >7 days despite optimal management; vomiting grade ⩾2; elevations in alanine aminotransferase (ALT) or aspartate aminotransferase (AST) levels grade ⩾2 together with elevated bilirubin levels grade >1; or any other AE grade 3/4. Dose reductions in the sunitinib group were indicated for patients with any AE grade 3/4. Treatment was discontinued if a third event occurred despite dose reductions. Where appropriate, patients were permitted to receive full supportive care, including transfusion of blood and blood products, and treatment with antibiotics, antiemetics, antidiarrhoeal agents, analgesics, erythropoietin or bisphosphonates. Additional chemo-, immuno-, radio- or hormone therapy was not permitted during the trial (with the exception of hormone replacement therapy). Palliative radiotherapy to control symptoms was permitted although radiated target lesions were no longer to be considered as target lesions.

Data in this report are for the analysis conducted 3 years after randomisation of the final patient, with a cut-off date of 21 February 2014. The study was still ongoing at the time of the cut-off.

### Endpoints and assessments

All efficacy parameters were assessed by the study investigators, without central review. The primary safety endpoint was change in QT interval from baseline to day 15 for nintedanib-treated patients (Eisen *et al*, 2013). The primary efficacy endpoint was investigator-assessed progression-free survival (PFS) at 9 months (defined as the proportion of patients without objective tumour progression (RECIST version 1.1) and alive 9 months after randomisation). Progression-free survival at 9 months was selected on the basis that the data available at the time that the study was designed suggested that the mean PFS for patients receiving sunitinib was ∼9 months (Motzer *et al*, 2006). Secondary efficacy endpoints included PFS, objective response (OR), duration of OR, overall survival (OS), time to treatment failure (TTF), time to progression (TTP) (Supplementary Table S2), and the pharmacokinetic characteristics at day 15. Disease assessments were performed based on tumour measurements evaluated according to RECIST version 1.1 (computed tomography or magnetic resonance imaging performed at baseline and every 12 weeks after treatment initiation), and graded by the investigator. The incidence and severity of AEs (according to Common Terminology Criteria for Adverse Events (CTCAE) version 3.0; Cancer Therapy Evaluation Program (2006)), and the laboratory parameters, vital signs, electrocardiogram (ECG) profiles and physical examination results were also assessed.

### Statistical analysis

The sample size in the nintedanib group was selected to ensure sufficient power to assess the primary safety endpoint. Using a paired *t*-test with a one-sided significance level of 0.05, a sample size of 60 patients had 90% power to reject the null hypothesis that the difference in QTcF means (post-treatment–baseline) would be >10 ms (i.e., above the regulatory threshold) in favour of the alternative hypothesis that the two means were equivalent, when the expected mean difference was 2.70 ms with a SD of 19 ms (Eisen *et al*, 2013). With 60 patients in the nintedanib group, an additional 30 patients in the sunitinib group provided a high probability of recording any numerically positive treatment effect on the primary efficacy endpoint (i.e., a greater proportion of patients with estimated 9-month PFS with nintedanib versus sunitinib) of ∼70%, although this was not derived from hypothesis testing. All analyses in this report were descriptive and exploratory in nature, and performed on the treated set (patients who received at least one dose of study treatment). A *post hoc* analysis also compared outcomes in the subpopulation of patients with bone or liver metastases at baseline, due to recent data showing a negative impact of these metastatic sites on survival in RCC for patients treated with targeted agents (Mckay *et al*, 2014).

The primary efficacy endpoint of 9-month PFS was derived from Kaplan–Meier (KM) estimates in order to take account of patients who were censored before 9 months (e.g. due to missing tumour assessments or switching to alternative therapy prior to progression) and compared between treatments using a normal approximation test. OR was assessed according to RECIST version 1.1. The number and percentage of responders was summarised along with the duration of response; the objective response rate was compared using a logistic regression model stratified by Motzer risk score and previous surgery for RCC. Additional efficacy endpoints of TTP, TTF and OS were evaluated using KM analysis; OS was calculated as the time to death in days. All patients were followed up for survival until death due to any cause, withdrawal of consent or 3 years following enrolment of the final subject. Progression-free survival and OS were analysed using a stratified log-rank test while HRs and CIs for PFS, TTP, TTF and OS were obtained from stratified Cox proportional hazards models; in both cases, the stratification factors were Motzer risk score and previous surgery for RCC. *Post hoc* analyses in the subpopulation of patients with bone or liver metastases were unstratified. *Post hoc* analyses of patient demographic and baseline characteristics, AEs reported in ⩾10% of patients and AEs associated with TKIs were performed using the Wilcoxon–Mann–Whitney test for continuous variables and χ^2^-based tests or exact tests, as appropriate, for categorical variables. For all results, *P*-values are reported for descriptive purposes only. Statistical analysis was performed using SAS version 9.2 (SAS Institute Inc., Cary, NC, USA) with the exception of baseline characteristics and AEs, for which version SAS version 9.4 was used.

## Results

### Patients

**Figure 1:**
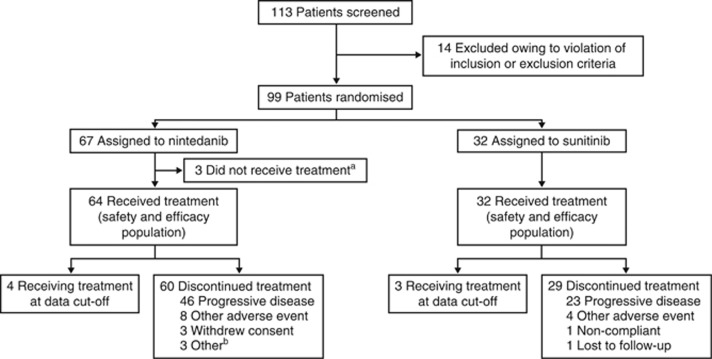
**Patient enrolment and study flow (CONSORT diagram).** ^a^One patient was randomised in error and had no measurable disease; one patient withdrew consent prior to receiving their first dose of study treatment; and one patient who was admitted with pleural effusion was not considered well enough to continue, and was withdrawn before receiving their first dose of study treatment. ^b^One patient discontinued treatment with a left ventricular fraction level below threshold, one patient discontinued with signs of clinical progression that were not confirmed, and a further one patient discontinued with evidence of increasing bone destruction of the right maxilla but no other sites of progression.

Of the 113 patients enrolled between 11 March 2010 and 14 December 2010, 99 were randomised and 96 received either nintedanib (*n*=64) or sunitinib (*n*=32) (Figure 1; Table 1). Three patients in the nintedanib group were not treated and were excluded from the safety and efficacy evaluations; reasons for withdrawal are outlined in Figure 1. Baseline characteristics were balanced (Table 1), and there was a male predominance in both groups (68.8%). Most patients had a favourable/intermediate MSKCC score (95.3% *vs* 93.8%), and had undergone prior nephrectomy (87.5% in both groups). The number of metastatic sites showed a similar pattern between the groups, but a higher proportion of patients in the nintedanib *vs* the sunitinib group had metastases in the bone (43.8% (*n*/*N*=28/64) *vs* 25.0% (8/32)) or liver (34.4% (22/64) *vs* 25.0% (8/32)).

### Treatment

As of the analysis cut-off date of 21 February 2014, 93.8% and 90.6% of patients treated with nintedanib and sunitinib, respectively, had discontinued treatment, whereas the remaining four (6.3%) and three (9.4%) patients in the nintedanib and sunitinib groups, respectively, were continuing treatment. Treatment discontinuation due to progressive disease occurred in 71.9% of patients, and treatment discontinuation owing to the other AEs occurred in 12.5% of patients, in both groups. The mean (median; range) duration of study treatment exposure was 348 (252; 22–1303) and 391 (239; 4–1334) days for patients in the nintedanib and sunitinib groups, respectively. Dose reductions occurred in 25.0% of patients in each treatment group, with a mean time to first dose reduction of 209 days with nintedanib and 186 days with sunitinib.

### Efficacy

**Figure 2:**
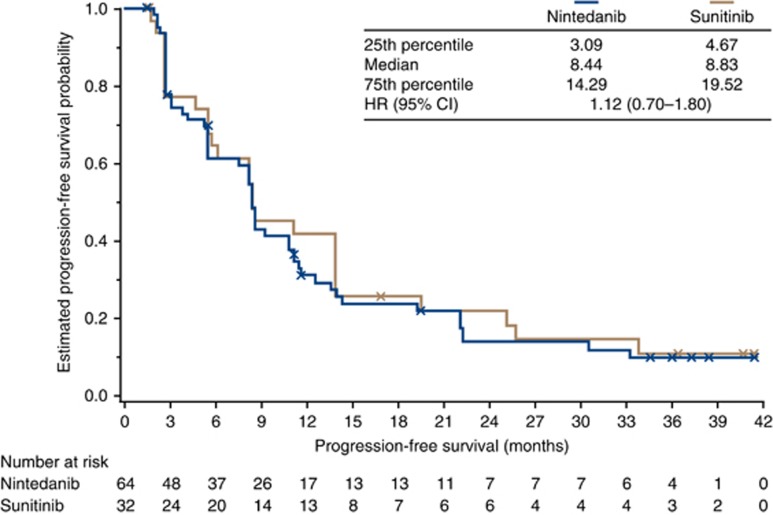
**Kaplan–Meier plot for progression-free survival by investigator assessment.** Patients without documented progression were censored at the date of their last tumour assessment. Crosses denote censoring events. At the cut-off date for 3-year analysis, 53 patients (82.8%) in the nintedanib group and 27 (84.4%) in the sunitinib group had progressed or died. Hazard ratio and confidence intervals for the overall treatment groups were obtained from Cox proportional hazards models stratified by Motzer risk score and previous surgery. Abbreviations: CI=confidence interval; HR=hazard ratio.

The primary efficacy endpoint of PFS at 9 months was 43.1% for patients treated with nintedanib and 45.2% for sunitinib, derived from KM estimates (*P*=0.85 for the intergroup comparison). *Post hoc* sub-analysis revealed PFS at 9 months for patients with liver or bone metastases at baseline to be 29.0% and 43.9%, respectively, with nintedanib and 14.3% and 25.0% with sunitinib. Median investigator-assessed PFS in the overall treatment groups was 8.4 months in each group (HR, 1.12; 95% CI: 0.70–1.80; *P*=0.64; Figure 2). Summaries of PFS for the patient subgroups defined by stratification factors (prior nephrectomy (yes/no) and MSKCC risk score (favourable–intermediate/poor) did not reveal any notable differences between groups. Median PFS for patients with liver metastases at baseline was 8.4 months with nintedanib and 5.6 months with sunitinib (HR, 0.43; 95% CI: 0.17–1.05; *P*=0.06). Progression-free survival was comparable in both treatment groups for patients with bone metastases at baseline. In the overall treatment groups, median TTP was 8.5 months (HR, 1.14; 95% CI: 0.70–1.87; *P*=0.60) and median TTF was 8.4 months (HR, 1.14; 95% CI: 0.72–1.81; *P*=0.57). At the cut-off date for this 3-year analysis, disease progression was recorded for 49 (76.6%) and 25 (78.1%) patients in the nintedanib and sunitinib groups, respectively, and treatment failure for 58 (90.6%) and 28 (87.5%) patients, respectively.

A confirmed OR was achieved by 20.3% and 31.3% of patients in the nintedanib and sunitinib groups, respectively (OR, 0.58; 95% CI: 0.22–1.51; *P*=0.26; Table 2). The median duration of OR was 19.4 months and 12.2 months in the nintedanib and sunitinib groups, respectively. Disease control was achieved by 76.6% and 78.1% of patients in the nintedanib and sunitinib groups, respectively. In patients with liver metastases at baseline, a confirmed all-disease site OR was achieved by 18.2% of patients with nintedanib and 12.5% with sunitinib. Similarly, in patients with bone metastases at baseline a confirmed all-disease site OR was achieved by 21.4% with nintedanib and 12.5% with sunitinib.

**Figure 3:**
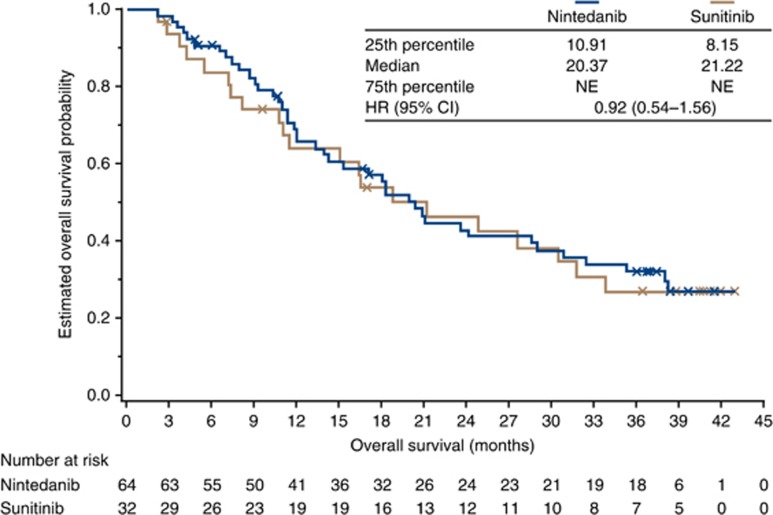
**Kaplan–Meier plot for overall survival.** Patients without documented death at the time of analysis were censored on the date that they were last known to have been alive. Crosses denote censoring events. At the cut-off date for 3-year analysis, 42 patients (65.6%) in the nintedanib group and 21 (65.6%) in the sunitinib group had died. Hazard ratio and confidence intervals for the overall treatment groups were obtained from Cox proportional hazards models stratified by Motzer risk score and previous surgery. Abbreviations: CI=confidence interval; HR=hazard ratio; NE=not evaluable.

At the cut-off date for the 3-year analysis, 42 (65.6%) and 21 patients (65.6%) in the nintedanib and sunitinib groups, respectively, had died. Median OS was 20.4 and 21.2 months for patients treated with nintedanib and sunitinib, respectively, with an HR of 0.92 (95% CI: 0.54–1.56; *P*=0.76; Figure 3). In the *post hoc* sub-analysis of patients with liver metastases at baseline, a median OS of 12.1 months was observed with nintedanib *vs* 8.2 months with sunitinib (HR, 0.50; 95% CI: 0.20–1.26; *P*=0.13). For patients with bone metastases at baseline, median OS was 18.3 months with nintedanib and 11.0 months with sunitinib (HR, 1.26; 95% CI: 0.43–3.72; *P*=0.68). Post-study anticancer therapy was more common in the nintedanib group (39.1% *vs* 25.0% Supplementary Table S3). The most common post-study anticancer therapy overall was everolimus (12.5% for both groups), but a markedly greater proportion of patients in the nintedanib *vs* sunitinib group received interferon (14.1% *vs* 3.1%).

### Safety

Overall incidence of any-grade AEs among patients treated with nintedanib and sunitinib was 90.6% and 93.8%, respectively (Table 3). Adverse events considered to be drug-related occurred in 75.0% of patients treated with nintedanib and 68.8% treated with sunitinib. Adverse events that were grade ⩾3 were reported in 43.8% and 59.4% of patients and serious AEs were reported in 31.3% and 34.4% of patients treated with nintedanib and sunitinib, respectively.

The most common AEs of any grade reported with nintedanib were diarrhoea, nausea and fatigue (Table 3). Adverse events occurring markedly more frequently in patients receiving nintedanib *vs* sunitinib were diarrhoea, and increases in gamma-glutamyltransferase (GGT), AST (any grade: 9.4% *vs* 0%) and ALT(7.8% *vs* 0%). Adverse events reported more frequently by patients receiving sunitinib *vs* nintedanib were: stomatitis, hand–foot syndrome (HFS), dyspepsia, anaemia, hypertension, hypothyroidism, dyspnoea and increased lipases.

The incidences of AEs associated with VEGF inhibitors were comparable between the nintedanib and sunitinib groups: thromboembolic events and haemorrhage (Table 4). Interestingly, the rates of hypertension (10.9% *vs* 15.6%) and hypothyroidism (3.1% *vs* 15.6%) were lower in the nintedanib arm than the sunitinib arm. There were no recorded events of gastrointestinal (GI) perforation in either group (Table 4).

Rates of patients with any-grade AEs associated with cardiac disorders were comparable with nintedanib and sunitinib (Table 4). All cardiac disorder events were grade 1 or 2, with the exception of one patient in each group with a myocardial infarction.

The proportion of patients with AEs (of any grade) in the nintedanib *vs* sunitinib groups were markedly lower for dermatologic AEs, such as HFS, which was not observed in nintedanib-treated patients (Table 4). Haematological AEs occurred more frequently in patients in the sunitinib group.

The most frequent AEs (⩾5%) leading to dose reduction in either group were diarrhoea (10.9% with nintedanib *vs* 6.3% with sunitinib), stomatitis (0.0% *vs* 9.4%), fatigue (0.0% *vs* 6.3%) and lethargy (0.0% *vs* 6.3%). Discontinuations due to AEs occurred in 17.2% and 15.6% of patients treated with nintedanib and sunitinib, respectively. Adverse events leading to death occurred for one patient in the nintedanib group (myocardial infarction and cerebrovascular accident) and one in the sunitinib group (ascites), which were not considered by the investigator to be drug-related.

Therapy discontinuation secondary to AEs, mean duration of treatment exposure, and dose reductions appeared similar for both the nintedanib and sunitinib groups. This suggests patient tolerability was similar for both drugs.

## Discussion

The overall efficacy of TKIs in renal cancer has been limited because of issues with drug resistance and toxicity (Conti *et al*, 2013). Sunitinib has become established as a standard-of-care for first-line treatment of metastatic RCC based on significant survival benefits in comparison with traditional cytokine-based therapy (Motzer *et al*, 2007, 2009). However, safety data from clinical trials and a sunitinib expanded access programme have demonstrated relatively high rates of AEs experienced by patients (Motzer *et al*, 2007, 2009; Gore *et al*, 2009). A recent observational study of clinical practice patterns reported that 10.3% of patients with RCC experienced a grade 3 or 4 AE over 24 weeks of first-line sunitinib therapy (Porta *et al*, 2014). The most frequent grade 3/4 AEs were fatigue, mucositis/stomatitis, diarrhoea, HFS, pain and thrombotic events. At 24 weeks, 31.8% of patients had experienced at least one sunitinib dose reduction, 20.3% at least one treatment interruption and 5.5% of patients had discontinued treatment. Overall, more than one third of patients required at least one treatment modification due to an AE in the first 24 weeks of treatment and there was a statistically significant inverse correlation between AEs and dose intensity. Importantly, a significant relationship was also observed between dose intensity and shorter survival, and between dose discontinuation and shorter survival. These data emphasise the importance of tolerability to efficacy outcomes for antiangiogenic TKIs in RCC.

In the current exploratory study, treatment with nintedanib provided an investigator-assessed 9-month PFS and median PFS and OS similar to sunitinib. These outcomes were greater with nintedanib than sunitinib in a *post hoc* sub-analysis of patients with liver metastases at baseline, although confirmation in a larger scale trial is required. Nevertheless, this observation is interesting given that the presence of metastases at these sites has a significant negative impact on survival in patients treated with targeted agents, including antiangiogenic agents (Mckay *et al*, 2014). The overall findings of this study are consistent with a study comparing first-line nintedanib with standard-of-care sorafenib in 95 Asian patients with advanced HCC (Cheng *et al*, 2015). In that study, efficacy was comparable between the groups, but nintedanib had a more favourable safety profile than sorafenib, with lower rates of AEs rated grade ⩾3 (56% *vs* 84%), serious AEs (46% *vs* 56%), AEs leading to dose reductions (19% *vs* 59%) and drug discontinuation (24% *vs* 34% Cheng *et al*, 2015). A similar study of 93 European patients with HCC also showed that efficacy with nintedanib was comparable to sorafenib and that nintedanib was associated with a manageable safety profile (Palmer *et al*, 2015).

In the current study, the most frequent any-grade AEs associated with nintedanib *vs* sunitinib were diarrhoea (62.5% *vs* 50.0%), nausea (37.5% *vs* 34.4%) and fatigue (25.0% *vs* 25.0%); however, the majority of these AEs were grade 1 or 2, and the incidence of grade ⩾3 events for nintedanib was comparable to that for sunitinib. The rate of patients with AEs of increased GGT, ALT and AST liver enzymes was higher with nintedanib than with sunitinib, while AEs of increased lipases were higher with sunitinib than for nintedanib.

Although treatment with antiangiogenic TKIs has been associated with certain specific AEs, including, bleeding, GI perforation and thromboembolism, nintedanib showed only moderate frequencies of hypertension, hypothyroidism and any cardiac AEs and there were no signs and symptoms for HFS, stomatitis or any haematological laboratory abnormalities.(Ravaud, 2011) Hypertension was once considered a potential indicator for efficacy of anti-VEGF therapy but it has not been validated as a predictive marker and a recent meta-analysis showed no association (Hurwitz *et al*, 2013). Furthermore, the frequency and severity of hypertension as an AE varies between studies, depending on the tumour type and other patient-related factors. Therefore, a moderate rate of hypertension with nintedanib does not indicate a lack of VEGFR inhibition. It should also be mentioned that the rather low incidences of these specific AEs are unlikely to reflect inadequate dosing: the pharmacokinetic characteristics of nintedanib in this study population (Eisen *et al*, 2013), were similar to those seen with nintedanib for other tumour types (Ellis *et al*, 2010; Okamoto *et al*, 2010; Doebele *et al*, 2012; Du Bois *et al*, 2013).

As previously reported, treatment with nintedanib in this study was not associated with QT prolongation (Eisen *et al*, 2013). Comparing ECG traces at baseline and day 15, slight increases in QTcF were seen at certain time points. However, the upper limits of the two-sided 90% CIs for the adjusted mean time-matched changes were well below the regulatory threshold of 10 ms at all times (Eisen *et al*, 2013). Safety data presented in this report also revealed a low rate of cardiovascular AEs in both groups. Therefore, the safety profile of nintedanib appears to be generally manageable by dose reductions and symptomatic treatment.

Limitations of this study were that it was a Phase II exploratory study not powered to detect a significant difference in efficacy parameters. Furthermore, patients and physicians were not blinded to the study treatment, which may have influenced outcomes, and no quality of life parameters were included to provide insight into the implications of differences between the treatment arms. Future studies should also include analysis of biomarkers to investigate the biological implications of FGFR inhibition in previously untreated patients and in patients previously treated with anti-VEGF therapy.

As an inhibitor of VEGFR, PDGFR and FGFR angiogenic signalling pathways, nintedanib may have the potential to overcome acquired resistance seen with other antiangiogenic TKIs. A recent Phase III study of the FGFR inhibitor dovitinib did not show a significant benefit over sorafenib in patients with metastatic RCC who had failed one prior VEGF pathway-targeted agent and one prior mammalian target of rapamycin (mTOR) inhibitor (Motzer *et al*, 2014). However, the vast majority of patients (92%) entered the study having received mTOR inhibitor therapy after VEGF-targeted therapy, and FGF2 levels were not increased at baseline. It is conceivable that FGF2 levels may have risen during VEGF pathway-targeted therapy, reflecting FGFR-mediated resistance, only to fall again during mTOR inhibitor therapy. Thus, further study of FGFR inhibitors that includes rigorous biomarker analysis is required to determine the true role of FGFR inhibitors in overcoming resistance to VEGF-targeted therapy.

## Conclusions

Previously reported data from this Phase II study demonstrated that there was no prolongation of the QTcF interval with nintedanib (Eisen *et al*, 2013). The results of the analysis reported here show promising efficacy for nintedanib in comparison with standard-of-care sunitinib as first-line treatment for patients with advanced RCC. The data also suggest that nintedanib has a manageable safety profile, with incidences of the AEs of hypertension, hypothyroidism, cardiac disorders, HFS and haematological abnormalities being lower than typically expected with the use of a TKI. A larger-scale trial with greater statistical power is required to confirm these efficacy and safety findings. Further evaluation of nintedanib in advanced RCC may be explored, particularly as second-line therapy after failure of TKIs, where there is a continuing unmet clinical need for new therapies that balance efficacy with a manageable safety profile.

## Figures and Tables

**Table 1:** Patient demographics and baseline characteristics for the treatment set

**Variable**	**Nintedanib (*****n*****=64)**	**Sunitinib (*****n*****=32)**
Median age, years (range)	62 (42–86)	58 (29–79)
**Sex, *n* (%)**		
Male	44 (68.8)	22 (68.8)
Female	20 (31.3)	10 (31.3)
Median BMI, kg/m^2^ (range)	27.6 (21.4–52.7)	28.0 (18.3–37.1)
**Country, *n* (%)**		
Hungary	2 (3.1)	0
Poland	14 (21.9)	6 (18.8)
Romania	5 (7.8)	0
Ukraine	33 (51.6)	18 (56.3)
UK	10 (15.6)	8 (25.0)
**Race**		
Caucasian	64 (100)	32 (100)
Median time since first diagnosis, years (range)	0.44 (0–11.8)	0.38 (0–9.1)
**ECOG performance status, *n* (%)**		
0	13 (20.3)	10 (31.3)
1	51 (79.7)	22 (68.8)
**MSKCC risk category, *n* (%)**		
Favourable/intermediate	61 (95.3)	30 (93.8)
Poor	3 (4.7)	2 (6.3)
**Nuclear clinical grading, *n* (%)**		
Not available	20 (31.3)	8 (25.0)
I	3 (4.7)	2 (6.3)
II	10 (15.6)	7 (21.9)
III	9 (14.1)	5 (15.6)
IV	22 (34.4)	10 (31.3)
**Metastatic sites^a^, *n* (%)**		
0–1	10 (15.6)	5 (15.6)
2–3	41 (64.1)	21 (65.6)
>3	12 (18.8)	6 (18.8)
**Location of metastases^a^, *n* (%)**		
Liver	22 (34.4)	8 (25.0)
Lung	45 (70.3)	22 (68.8)
Bone	28 (43.8)	8 (25.0)
Brain	0	0
Adrenal	13 (20.3)	6 (18.8)
Other	45 (70.3)	28 (87.5)
**Prior nephrectomy, *n* (%)**		
No	8 (12.5)	4 (12.5)
Yes	56 (87.5)	28 (87.5)
**Concomitant disease, *n* (%)**		
Cardiovascular	22 (34.4)	10 (31.3)
Hypertension	23 (35.9)	12 (37.5)

Abbreviations: BMI=body mass index; ECOG=Eastern Oncology Cooperative Group; MSKCC=Memorial Sloan-Kettering Cancer Center.

There were no statistically significant differences between the two groups at baseline on the basis of Wilcoxon–Mann–Whitney tests for continuous variables and χ^2^ based tests or exact tests, as appropriate, for categorical variables.

a Data missing for one patient in the nintedanib treatment group.

**Table 2:** Confirmed best tumour response and disease control according to RECIST version 1.1 criteria

**Variable, *n* (%)**	**Nintedanib (*****n*****=64)**	**Sunitinib (*****n*****=32)**
Disease control	49 (76.6)	25 (78.1)
Objective response	13 (20.3)	10 (31.3)
**Confirmed best tumour response**		
Complete response	0 (0.0)	1 (3.1)
Partial response	13 (20.3)	9 (28.1)
Stable disease	36 (56.3)	15 (46.9)
Progressive disease	14 (21.9)	5 (15.6)
Not evaluable	1 (1.6)	2 (6.3)

Abbreviation: RECIST=Response Evaluation Criteria in Solid Tumors.

**Table 3:** Summary of adverse events^a^ reported in ⩾10% of patients in either treatment arm

	**Nintedanib (*****n*****=64)**		**Sunitinib (*****n*****=32)**	
**MedDRA preferred term, *n* (%)**	**Any grade**	**Grade ⩾3**	**Any grade**	**Grade ⩾3**
Patients with any serious AE	20 (31.3)	16 (25.0)	11 (34.4)	8 (25.0)
Patients with any AE	58 (90.6)	31 (48.4)	30 (93.8)	19 (59.4)
**Type of AE**				
Diarrhoea	40 (62.5)	2 (3.1)	16 (50.0)	1 (3.1)
Nausea	24 (37.5)	0 (0.0)	11 (34.4)^b^	1 (3.1)
Fatigue	16 (25.0)	2 (3.1)	8 (25.0)	2 (6.3)
Vomiting	10 (15.6)	0	7 (21.9)	1 (3.1)
Decreased appetite	10 (15.6)	0	6 (18.8)	0
Increased GGT	8 (12.5)	7 (10.9)	1 (3.1)	1 (3.1)
Decreased weight	8 (12.5)	1 (1.6)	2 (6.3)	0
Stomatitis^c^	0	0	10 (31.3)	2 (6.3)
Hand–foot syndrome^c^	0	0	10 (31.3)	0
Dyspepsia^c^	2 (3.1)	0	7 (21.9)	0
Anaemia	4 (6.3)	0	5 (15.6)	2 (6.3)
Hypertension^c^	2 (3.1)	0	5 (15.6)	2 (6.3)
Hypothyroidism^c^	2 (3.1)	0	5 (15.6)	0
Constipation	5 (7.8)	0	4 (12.5)	0
Dysgeusia	3 (4.7)	0	4 (12.5)	0
Increased lipase	2 (3.1)	2 (3.1)	4 (12.5)	3 (9.4)
Dyspnoea^c^	1 (1.6)	0	4 (12.5)	0

Abbreviations: AE=adverse event; GGT=gamma-glutamyltransferase; MedDRA=Medical Dictionary for Regulatory Activities.

a Classified by Common Terminology Criteria for Adverse Events (CTCAE) version 3.0.

b One patient reported nausea, but their CTCAE grade was not recorded.

c *P*<0.05 for comparison of any grade AEs based on *χ*^2^-tests or Suissa–Shuster exact tests as appropriate.

**Table 4:** Summary of adverse events associated with TKIs

	**Nintedanib (*****n*****=64)**		**Sunitinib (*****n*****=32)**	
**Standardised MedDRA Query, *n* (%)**	**Any grade**	**Grade ⩾3**	**Any grade**	**Grade ⩾3**
**Class effects of VEGF inhibitors**				
Hypertension	7 (10.9)	1 (1.6)	5 (15.6)	2 (6.3)
Haemorrhage	7 (10.9)	2 (3.1)	4 (12.5)	2 (6.3)
Thromboembolic events	4 (6.3)	3 (4.7)	1 (3.1)	1 (3.1)
GI perforation	0	0	0	0
Hypothyroidisma,^b^	2 (3.1)	0	5 (15.6)	0
**Dermatological AEs**				
Cutaneous serious skin reactions	0	0	12 (37.5)	3 (9.4)
Stomatitisb,^c^	0	0	10 (31.3)	2 (6.3)
Hand–foot syndromeb,^c^	0	0	10 (31.3)	0
Rasha,^b^	1 (1.6)	0	5 (15.6)	1 (3.1)
**Cardiac AEs**				
Cardiac arrhythmia	8 (12.5)	0	6 (18.8)	0
Myocardial infarction^c^	1 (1.6)	1 (1.6)	1 (3.1)	1 (3.1)
Cardiac failure	2 (3.1)	0	1 (3.1)	0
**Haematological AEs**				
Anaemia^c^	4 (6.3)	0	5 (15.6)	2 (6.3)
Neutropeniab,^c^	0	0	3 (9.4)	0
Thrombocytopenia^c^	0	0	2 (6.3)	2 (6.3)

Abbreviations: AE=adverse event; GI=gastrointestinal; TKI=tyrosine kinase inhibitor.

a Tailored special search category.

b *P*<0.05 for comparison of any grade AEs based on Suissa–Shuster exact tests.

c MedDRA preferred term.

## Notes

TE is a part-time employee (from 1 September 2014) of AstraZeneca and owns stock or other ownership interest in the company, and has had a consulting or advisory role and received honoraria from Bayer, Pfizer, AVEO Oncology and GlaxoSmithKline. RJJ has received honoraria from Bristol-Myers Squibb, GlaxoSmithKline, Novartis, Pfizer and Roche. A-BL, GT and HD are all employees of Boehringer Ingelheim. IB and NM report no conflicts of interest.
