# Supplementary material for: A randomised, phase II study of nintedanib or sunitinib in previously untreated patients with advanced renal cell cancer: 3-year results
Source: Br J Cancer. 2015 Oct 8;113(8):1140–7. doi: 10.1038/bjc.2015.313 (PMC4647871; doi:10.1038/bjc.2015.313)
Supplement: Supplementary Tables [file bjc2015313x1.doc]

Supplementary information

**Table S1. Patient eligibility criteria**

| Inclusion criteria   1. **Patients with unresectable or metastatic RCC, who had received no previous systemic anti-cancer treatment** 2. **Histologically-confirmed diagnosis of RCC with clear cell component** 3. **Age ≥18 years** 4. **Eastern Cooperative Oncology Group Performance Score 0 or 1** 5. **Life expectancy of ≥3 months** 6. **Measurable disease according to Response Evaluation Criteria in Solid Tumors (RECIST), i.e., presence of at least one target lesion according to RECIST criteria in a previously non-irradiated area** 7. **Serum creatinine <2 x upper limit of normal (ULN)** 8. **Written informed consent consistent with ICH-GCP guidelines** |
| --- |
| Exclusion criteria   1. **Serious illness or concomitant non-oncological disease such as neurologic, psychiatric, infectious disease, or active ulcers (gastro-intestinal tract, skin), or laboratory abnormality that might increase the risk associated with trial participation or trial drug administration and in the judgment of the investigator would make the patient inappropriate for entry into the trial** 2. **Major injuries, bone fracture, and/or surgery within past 4 weeks, and/or planned surgical procedures during the trial period** 3. **Hypersensitivity to nintedanib, sunitinib, or the excipients of the trial drugs** 4. **Significant cardiovascular diseases (i.e., uncontrolled hypertension, unstable angina, history of infarction within past 12 months, congestive heart failure > New York Heart Association (NYHA) II, serious cardiac arrhythmia)** 5. **Left ventricular ejection fraction (LVEF) by echocardiography below local limits of normal** 6. **Hepatic function: total bilirubin outside of normal limits; ALT and AST >1.5 x ULN in patients without liver metastasis. For patients with liver metastasis: total bilirubin outside of normal limits, ALT and AST >2.5 x ULN** 7. **Coagulation parameters: international normalised ratio (INR) >2, prothrombin time (PT) and partial thromboplastin time (PTT) >50% of deviation of institutional ULN** 8. **Absolute neutrophil count (ANC) <1500/mL, platelets <100000/mL, haemoglobin <9.0 g/dL** 9. **History of clinically significant haemorrhagic or thromboembolic event in the past 6 months** 10. **Known inherited predisposition to bleeds or to thrombosis** 11. **History of clinically significant haemoptysis within the last 3 months (more than 1 tea-spoon of fresh blood per day)** 12. **Therapeutic anticoagulation (except low-dose heparin and/or heparin flush as needed for maintenance of an indwelling intravenous device) or antiplatelet therapy (except for lowdose therapy with acetylsalicylic acid <325 mg/day)** 13. **Previous treatment for RCC with targeted agents (in particular including any antibody and any VEGF/VEGFR, epidermal growth factor receptor [EGFR], and mechanistic target of rapamycin] inhibitors [mTOR]), immunotherapy (interferon [IFNα] or interleukin-2 [IL-2]) or chemotherapy** 14. **Treatment with other investigational drugs or participation in another clinical trial within the past 4 weeks before start of therapy or concomitantly with this trial** 15. **Patients unable to comply with the protocol** 16. **Pregnancy or breast feeding** 17. **Active alcohol or drug abuse** 18. **Women of childbearing potential, or men who were able to father a child, unwilling to use a medically acceptable form of contraception during the trial period** 19. **Symptomatic central nervous system (CNS) metastatis or leptomeningeal disease as documented by CT, MRI, or analysis of cerebrospinal fluid requiring radiotherapy, steroids, or anticonvulsive treatment** 20. **Radiotherapy within the previous 4 weeks** 21. **QTcF interval >500 ms at screening** |

Table S2. Definition of efficacy endpoints

| **Efficacy endpoint** | **Definition** |
| --- | --- |
| **Primary efficacy endpoint** |  |
| Progression-free survival at 9 months | Derived from the 9 month time-point of Kaplan-Meier curves produced for the overall PFS |
| **Secondary efficacy endpoints** |  |
| Progression-free survival | Time from randomization to the occurrence of disease progression (determined by investigator evaluation according to RECIST version 1.1) or death, whichever occurred first. Patients without documented progression were censored at the date of their final evaluable imaging |
| Objective response | Complete response or partial response as determined by RECIST version 1.1 |
| Duration of objective response | Time from the first objective response to the time of disease progression (determined by RECIST version 1.1) or death, whichever occurred first |
| Overall survival | Time from randomization to death. Patients for whom there was no evidence of death at the time of analysis were censored on the date that they were last known to be alive |
| Time to progression | Time of randomization to time of disease progression (by RECIST version 1.1). Patients with no progression were censored at the date of their last evaluable imaging |
| Time to treatment failure | Time of randomization to the time of disease progression (by RECIST version 1.1), global deterioration of health status requiring treatment discontinuation, death or start of a new anticancer treatment, whichever came first. Patients with no treatment failure were censored at the date of their last evaluable imaging |

Abbreviations: RECIST = Response Evaluation Criteria in Solid Tumors.

**Table S3. Summary of post-study anticancer therapy**

| **n (%)^a^** | Nintedanib (n=64) | Sunitinib (n=32) |
| --- | --- | --- |
| **Patients with disease progression** | **49 (76.6%)** | **25 (78.1%)** |
| **Patients who received post-study anticancer therapy** | **25 (39.1%)** | **8 (25.0%)** |
| **Interferon** | **9 (14.1%)** | **1 (3.1%)** |
| **Everolimus** | **8 (12.5%)** | **4 (12.5%)** |
| **Pazopanib** | **5 (7.8%)** | **0** |
| **Sunitinib** | **3 (4.7%)** | **1 (3.1%)** |
| **Radiotherapy** | **3 (4.7%)** | **0** |
| **Investigational agent** | **2 (3.1%)** | **1 (3.1%)** |
| **Interleukin-2** | **2 (3.1%)** | **0** |
| **Tamoxifen** | **2 (3.1%)** | **0** |
| **Capecitabine** | **0** | **1 (3.1%)** |
| **Cediranib or cediranib plus saracatinib^b^** | **0** | **1 (3.1%)** |
| **Lomustine** | **1 (1.6%)** | **0** |
| **Temsirolimus** | **1 (1.6%)** | **0** |
| **Vinorelbine** | **1 (1.6%)** | **0** |

**^a^Patients may have received more than one anticancer therapy**.

**^b^Randomized treatment in a double-blind study.**
